# Laser-induced forward transfer based laser bioprinting in biomedical applications

**DOI:** 10.3389/fbioe.2023.1255782

**Published:** 2023-08-21

**Authors:** Jinlong Chang, Xuming Sun

**Affiliations:** ^1^ School of Medical Engineering, Xinxiang Medical University, Xinxiang, China; ^2^ Xinxiang Key Laboratory of Neurobiosensor, Xinxiang Medical University, Xinxiang, China

**Keywords:** 3D printing, bioprinting, laser-induced forward transfer, tissue engineering, biomedical applications

## Abstract

Bioprinting is an emerging field that utilizes 3D printing technology to fabricate intricate biological structures, including tissues and organs. Among the various promising bioprinting techniques, laser-induced forward transfer (LIFT) stands out by employing a laser to precisely transfer cells or bioinks onto a substrate, enabling the creation of complex 3D architectures with characteristics of high printing precision, enhanced cell viability, and excellent technical adaptability. This technology has found extensive applications in the production of biomolecular microarrays and biological structures, demonstrating significant potential in tissue engineering. This review briefly introduces the experimental setup, bioink ejection mechanisms, and parameters relevant to LIFT bioprinting. Furthermore, it presents a detailed summary of both conventional and cutting-edge applications of LIFT in fabricating biomolecule microarrays and various tissues, such as skin, blood vessels and bone. Additionally, the review addresses the existing challenges in this field and provides corresponding suggestions. By contributing to the ongoing development of this field, this review aims to inspire further research on the utilization of LIFT-based bioprinting in biomedical applications.

## 1 Introduction

3D printing is a technology that was first introduced by Charles Hull, which allows for the precise manufacturing of pre-designed 3D structures in a bottom-up and layer-by-layer fashion using digital computer-aided design (Miller and Burdick, 2016; Shahrubudin et al., 2019). Due to its high geometric precision, 3D printing has been widely used in various fields such as design, automotive, medical, and architecture, enabling the manufacturing of 3D structures with complex internal structures and unique external features (Shahrubudin et al., 2019). In the biomedical field, 3D printing is commonly referred to as bioprinting and has been applied in areas such as tissue engineering, drug discovery, and regenerative medicine (Yusupov et al., 2020; Wu et al., 2022).

Bioprinting is a novel research field that involves the integration of biological materials into 3D printing for rapid prototyping manufacturing (Fu et al., 2022; Wu et al., 2022). It utilizes the technology of additive manufacturing to create intricate three-dimensional structures by depositing biological materials on a receiver base or a substrate. The technology enables the precise placement of seed cells, biological materials, and biomolecules in space to generate tissue or organ substitutes that exhibit biological activity, closely resembling or even surpassing the functionality of target tissues or organs, thereby addressing various human health issues (Ayan et al., 2020; Mota et al., 2020; Daly et al., 2021; Dey et al., 2022). Bioprinting has successfully produced live tissues and organs, such as bones (Moncal et al., 2023), cartilage (Antich et al., 2020), blood vessels (Zhou et al., 2020), skin (Jin et al., 2021), nerves (Liu et al., 2021), and even artificial ears (Bhattacharyya et al., 2023).

To construct three-dimensional biological models that mimic natural tissues or organs within the body, different techniques have been developed, including bioprinting and microfluidic devices. Among these methods, 3D bioprinting is the most creative, with the potential to create functional tissues that can bridge the gap between artificial engineered tissues and native tissues. Bioprinting can be divided into various types (Murphy and Atala, 2014; Gao et al., 2021), including inkjet-based bioprinting, extrusion-based bioprinting, and laser-assisted bioprinting (Foyt et al., 2018). Inkjet bioprinters use biological materials as printing materials (Li et al., 2020; Suntornnond et al., 2022). The print head is controlled by an electrically operated lifting platform, and droplets in the print head can be dropped and formed with the help of heat or sound waves to achieve the printing of a three-dimensional structure. Extrusion-based bioprinters consist of syringe, nozzle, and pressure system, which extrudes the bio-ink from the nozzle by means of a piston or screw mechanism utilizing the mechanical force or pneumatic pressure (Pati et al., 2016). The print head deposits the material at specified locations according to computer data-controlled printing paths to create the desired shape. The working principle of laser-assisted bioprinting is to concentrate laser energy on an absorbent layer on a glass plate, which pushes the biological ink to the receiving substrate in the form of a high-pressure liquid bubble (Dou et al., 2021; Yang et al., 2021). Laser-assisted printing has the advantages of an open print head, which eliminates clogging problems, as well as high resolution and the ability to print complex structures and high-viscosity (high-cell-density) materials.

Laser-induced forward transfer (LIFT) is a type of laser-assisted printing technology that was first proposed by Bohandy in 1986 (Bohandy et al., 1986). In 2004, it was first applied to bioprinting and successfully printed cell patterns with high cell viability (Barron et al., 2004). Compared with inkjet and extrusion printing methods, LIFT technology boasts high printing accuracy and resolution (up to the micron level), high throughput, and high cell survival rate (Kanaki et al., 2022). Since there is no need for any nozzles during the printing process and there is no issue of ink clogging during printing. Additionally, this method can be combined with other bioprinting technologies to expand its printing capabilities and has the ability for *in-situ* printing (Kérourédan et al., 2019). As a result, LIFT has been widely applied in bioprinting of drug (Kanaki et al., 2023), DNA (Colina et al., 2005), proteins (Paris et al., 2022), human osteosarcoma cells (Barron et al., 2005), human endothelial cells (Gaebel et al., 2011; Koch et al., 2021) and mesenchymal stem cells (Keriquel et al., 2017) with applications in drug delivery and testing, nucleic acid microarrays, protein microarrays, printing of cells, tissues and organs, as summarized in Table 1. The high cost of LIFT technology is a significant factor limiting its research and commercial application. However, with the increasing demand for the manufacturing of complex biological structures, the cost of laser-assisted printing technology is rapidly decreasing. At the same time, the cost of lasers is expected to further decrease, which may lead to a further reduction in the cost of this technology.

**TABLE 1:** Summary of the applications of LIFT in bioprinting.

Material transferred	Applications	References
Gemcitabine	Drug delivery and testing	(Kanaki et al., 2022)
Cisplatin	Drug delivery and testing	(Kanaki et al., 2023)
cDNAs	DNA microarrays	(Serra et al., 2004)
ssDNA oligomers	Capacitive microarray	(Tsekenis et al., 2012)
Peptides	Microarrays for infectious disease screening	(Paris et al., 2022)
Fibroblasts and keratinocytes	Skin regeneration	(Michael et al., 2013)
Human dermal fibroblasts	Skin repair	(Douillet et al., 2022)
Human umbilical vein endothelial cells	Microvascular networks	(Pirlo et al., 2012)
Endothelial cells	Capillary vessels	(Koch et al., 2021)
Human stem cells	3D corneal mimicking tissues	(Sorkio et al., 2018)
Human stem cells and endothelial cells	Cardiac regeneration	(Gaebel et al., 2011)
Mesenchymal stem cells	Cardiomyocyte construct	(Ma et al., 2013)
Human osteoprogenitors	Bone tissue biofabrication	Catros et al. (2011b)
Mesenchymal stem cells	Bone regeneration	(Keriquel et al., 2017)
Dorsal root ganglion neurons	Neuronal proliferation	(Curley et al., 2016)

This review focuses the biomedical application of bioprinting based on LIFT technology. The principle and ejection mechanisms of LIFT are briefly introduced. The various parameters related to LIFT bioprinting including laser energy, laser spot size, physical properties of the bioink, and thickness of the absorbing layer are discussed for effective and efficient bioprinting. We introduce the biomedical application of LIFT in printing of biomolecule microarrays cells, tissues, and organs. In addition, this review discusses the existing challenges and provides suggestions for future research.

## 2 Laser-induced forward transfer

**FIGURE 1:**
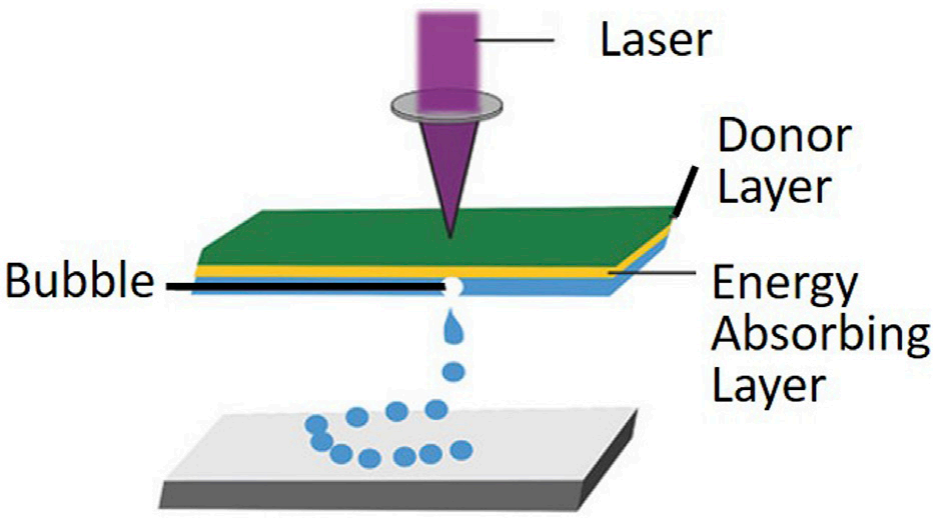
Laser-assisted bioprinting utilizes laser-induced rapid heating of the donor layer to form a bubble and propel the bioink onto the substrate. (from ref. (Foyt et al., 2018). licensed under Creative Commons Attribution 4.0 license).

The principle of LIFT operation involves focusing a beam of light through a transparent substrate onto a metal or polymer film, where some of the light is absorbed and converted into internal energy (Delaporte and Alloncle, 2016; Mota et al., 2020). This process increases the temperature, expands and deforms, and may even cause liquefaction or vaporization, leading to material transfer (Serra and Pique, 2019; Fernández-Pradas and Serra, 2020). The LIFT device mainly consists of a laser source, a donor with several layers, and a receiving substrate as shown in Figure 1. The laser source is typically a single-wavelength, single-mode pulsed laser. The donor includes a transparent substrate, usually a quartz slide with almost zero absorption of laser light, a laser absorber layer coated with metal or metal oxide, and a biological solution coating layer that contains biological materials such as DNA, proteins, or cells. The receiving substrate is a buffer-coated slide.

### 2.1 Laser source

As the heat source in LIFT, pulsed laser systems with pulse durations of a few nanoseconds are the most commonly used laser sources, although ultrafast laser systems with picosecond and femtosecond pulses can also be used (Fernández-Pradas and Serra, 2020; Yusupov et al., 2020). Optical elements such as beam splitters and lenses are used to modify, guide, and focus the laser beam onto the donor substrate-donor layer interface. The laser wavelength needs to match the transparency of the donor substrate and the absorption capacity of the donor layer, although it may not play a key role in the process. Additionally, laser system parameters such as laser energy density, pulse duration, pulse frequency, and pulse energy have a significant impact on the printing process and results. The choice of wavelength depends on the interacting material (intermediate layer or material to be transferred), with ultraviolet radiation being very common. In the LIFT bioprinting process, pulse energy and laser beam size are crucial parameters, while some studies have also identified laser fluence (proportional to pulse energy divided by spot area) as a key parameter (Boutopoulos et al., 2014).

### 2.2 Donor

The donor layer in LIFT bioprinting usually consists of three components: the substrate, the absorber layer, and the biomaterial layer (Morales et al., 2018). Transparent glass is commonly used as the substrate for near-infrared or visible laser wavelengths, while quartz and fused silica are used for ultraviolet wavelengths. Additionally, flexible organic substrates are being explored as potential substrates. The biomaterial layer serves as the “ink”, that is, printed, and the cell ink comprises cells, cell culture medium, and matrix material. The “ink” requires biochemical properties similar to those of native extracellular matrix, and typically contains cell medium (Vinson et al., 2017), glycerol (Guillotin et al., 2010) or fibrinogen (Koch et al., 2018), etc. The matrix material should closely mimic the structure and composition of the extracellular matrix, with excellent biocompatibility, molding properties, low cell toxicity, and ease of degradation. An absorber layer is typically added between the transparent substrate and the donor layer to prevent direct laser interaction with the material to be transferred. Ti, TiO_2_, or Au is commonly used as the metal absorber layer (Barron et al., 2004; Duocastella et al., 2010; Dou et al., 2021). Various types of UV-absorbing layers, such as polymer films (Fernández-Pradas and Serra, 2020), have also demonstrated similar printing outcomes.

### 2.3 Receiving substrate

The receiving substrate, typically a cover glass, is positioned parallel to the donor and placed on a 3D movable platform, along with the donor, to receive the printed biological droplets (Serra and Pique, 2019). The glass substrate is coated with a hydrogel layer or other biocompatible material, which plays a crucial role in maintaining the viability of the biomaterial after printing (Catros et al., 2011a). Firstly, the layer acts as a cushion, effectively reducing shear damage caused by the impact of the printed biological material on the substrate. Secondly, the hydrogel hydrates the biomaterial, preventing the evaporation of small droplets on the receiving substrate. Thirdly, the collagen and laminin contained in hydrogel also facilitates the adhesion of the printed organisms to the substrate and support their continuous differentiation. For instance, Ringeisen et al. observed that the thickness of the coating on the receiving substrate influenced the cellular activity during the printing of multifunctional embryonic cancer cells (Ringeisen et al., 2004). In the absence of any buffering substance, the activity of the printed cells was 5%. Meanwhile, when the coating thickness was increased from 20 to 40 μm, the cell activity improved from 50% to >95%. However, the optimal thickness of the coating layer varies depending on experimental conditions, such as the viscosity of the bioink, laser energy, and spot size.

## 3 Ejection mechanisms

LIFT is based on the principle of interaction between light and matter, where a portion of light is absorbed by a metal or polymer film of the donor and converted into internal energy, causing temperature increase and bubble formation when a laser beam is focused on the film through a transparent substrate (Serra and Pique, 2019; Dou et al., 2021). Subsequently, the bubbles expand and deform, leading to their collapse and the formation of a jet or droplet of bio-ink, which results in the transfer of material and ultimately the printing of cells or other biological materials in the bio-ink (Morales et al., 2018; Fernández-Pradas and Serra, 2020).

In order to explore the theoretical process of material transfer in LIFT, researchers have used numerical analysis and simulation methods to investigate the heat generated, temperature distribution, and material transfer mechanisms of different materials under different laser energies and pulse widths (Bohandy et al., 1988; Hu et al., 2017; Kalaitzis et al., 2019). To this end, they have proposed various theoretical models, including explosive ejection, phase-change ejection, and shockwave ejection. 1) The explosive ejection theory suggests that material transfer is caused by the pressure generated by laser ablation and vaporization, resulting in an explosion (Adrian et al., 1987). When the melting interface has not yet reached the air interface, the material has already undergone ablation and vaporization, and the gas pressure causes the material to undergo explosive ejection transfer in a very small space. 2) The phase-change ejection theory can explain the ejection of metal microdroplets well (Willis and Grosu, 2005). According to this theory, although metal materials expand under laser irradiation, they do not detach because they are constantly constrained by the solid state. At the same time, the focused laser gradually causes the melting interface to move along the metal film towards the air interface, until the intersection between the film and air is also melted, and the fully liquefied film at the intersection is no longer constrained, forming a metal droplet, that is, ejected and transferred. 3) The shockwave theory suggests that after heating, the film partially liquefies and vaporizes, and at the same time, the film tends to expand towards the substrate (Sotrop et al., 2013). When it reaches the substrate interface, a shockwave is generated, which causes the film to deform beyond its material strength and be ejected and transferred. However, these models generally only match well with their own experiments. Therefore, further research is needed to find a comprehensive model that can adequately explain the process of bubble formation and jet development in LIFT bioprinting.

Further research and understanding of LIFT theory is crucial to improve printing efficiency and accuracy, as well as to enhance the vitality of printed cells. As the formation of bubbles and jets is a complex multi-physics process, more in-depth studies are needed to improve the fundamental understanding of the interaction between laser and bio-ink, and the transfer process of bio-materials.

## 4 Parameters related to LIFT bioprinting

Several key factors, such as the formation of bubbles, jet development, deposition volume, resolution, and cell viability during the LIFT bioprinting process are influenced by various parameters (Serra and Pique, 2019; Dou et al., 2021), including 1) laser energy, 2) laser spot size, 3) physical properties of the bioink, 4) thickness of the absorbing layer.

### 4.1 Laser energy

**FIGURE 2:**
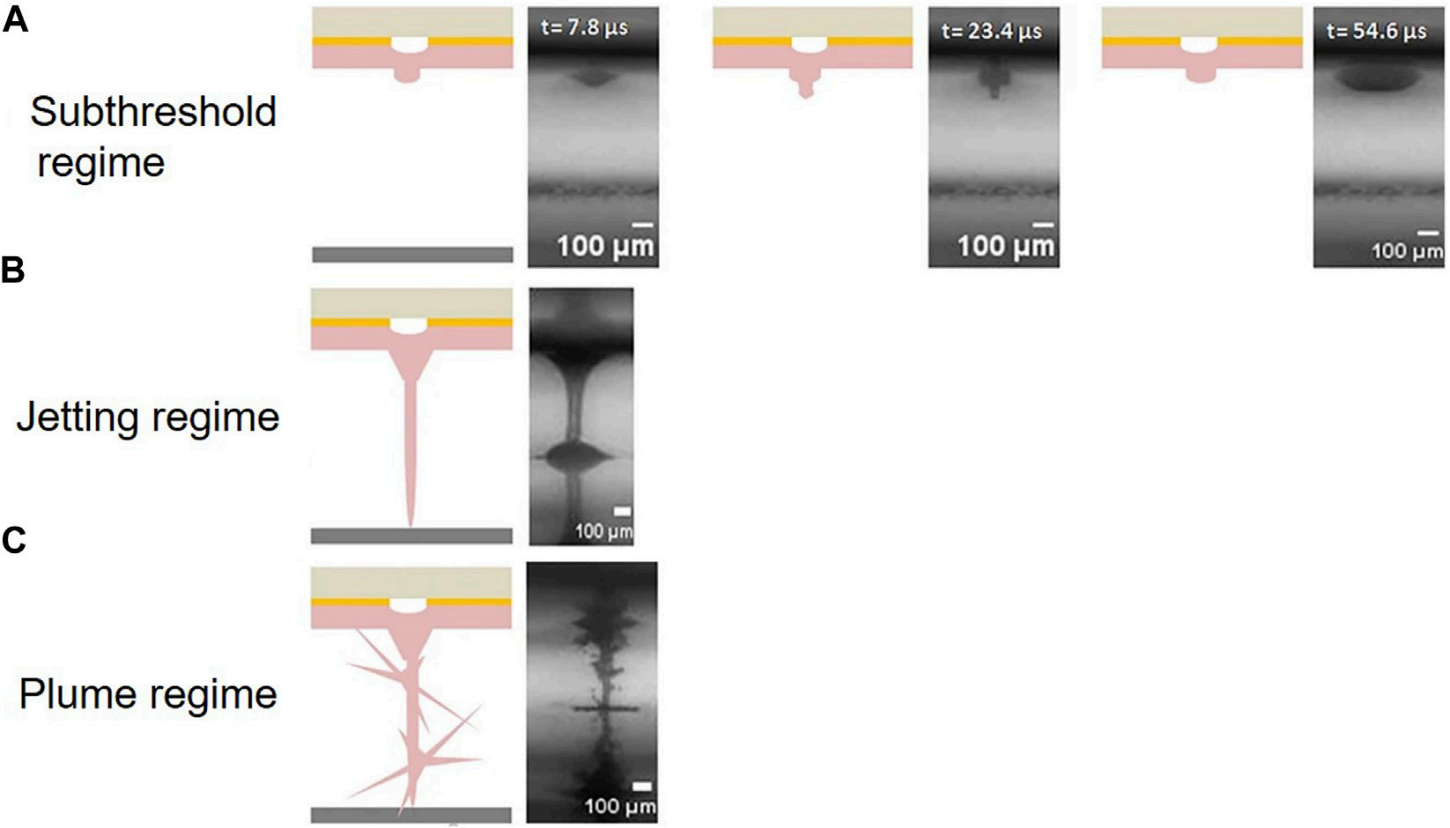
Schematic of three different regimes depending on the level of laser pulse energy: **(A)** sub-threshold regime, **(B)** jetting regime, and **(C)** plume regime. (from ref. (Kryou and Zergioti, 2022). licensed under Creative Commons Attribution 4.0 license).

The formation of the jet during LIFT bioprinting occurs in three different regimes depending on the level of laser pulse energy: the sub-threshold regime, jetting regime, and plume regime as shown in Figure 2 (Kryou and Zergioti, 2022). In the sub-threshold regime, the jet cannot form completely due to insufficient laser energy or high liquid viscosity, resulting in no material transfer. Conversely, in the plume regime, an unstable jet can occur due to excessive laser energy or low liquid density, leading to the formation of undesirable plumes and irregular droplets of varying sizes. Only when the laser energy is between the jetting and plume thresholds does stable jetting occur, enabling effective and controlled transfer of the bioink.

### 4.2 Laser spot size

The laser spot size is another important parameter that affects LIFT bioprinting and will determine the resolution of the print (Yusupov et al., 2020; Kanaki et al., 2022). A smaller laser spot will usually give a higher resolution, but it will also transfer less ink when printing and therefore result in poor print efficiency. Therefore, the right laser spot needs to be selected to balance resolution and print efficiency.

### 4.3 The viscosity of the bioink

It is worth noting that viscosity is not only an important indicator of the bioink, but also plays a crucial role during bioprinting (Barron et al., 2004; Kalaitzis et al., 2019). If the viscosity of the bioink is too low, splashing may occur during the printing process. While the viscosity is too high on the contrary, the laser may require a higher energy to trigger the ink jetting process. In other words, only when the ink has an appropriate viscosity, can a stable jet be formed. Similar to laser spot size, the viscosity of bioink also significantly affects printing resolution, especially which has a greater impact on resolution than laser energy at low laser energy.

### 4.4 The presence of cells

The presence of cells in bioink is a crucial consideration, as it can significantly impact the quality of LIFT bioprinting (Dou et al., 2021). Compared to printing with cell-free bioink, the inclusion of cells typically requires higher laser energy, resulting in slower jet speeds and smaller print spots (Zhang et al., 2017). Additionally, the non-uniform distribution of cells due to aggregation in the bioink can cause two types of non-ideal jetting behaviors during printing: non-straight jets with non-straight trajectories and straight jets with non-straight trajectories.

As mentioned before, the thickness of the absorber layer and the distance between the donor and receiver layers can also impact the success of the printing process. Therefore, it is essential to utilize appropriate parameters when employing LIFT technology for laser bioprinting. Doing so can help to maintain printing speed and prevent biological alterations, such as damage to the phenotypic or nucleic acid integrity of the cells during LIFT bioprinting.

## 5 Biomedical applications

### 5.1 LIFT printing of biomolecule microarrays

Microarray is a solid-phase-based technology used for high-throughput parallel detection and analysis of biomolecules (Sun et al., 2007; Chi et al., 2023). It enables simultaneous detection of thousands to millions of biomolecules, such as DNA, RNA, and proteins. The wide application of biological microarray includes gene expression analysis, genotyping, proteome-wide molecular interactions, and disease diagnosis. The fabrication of biological microarrays mainly rely on techniques such as spotting with photolithography, inkjet printing, and mechanical micro-spotting. Although these methods are relatively mature, each approach has at least one limitation in regards to post-printing biomolecular activity, the molecule spot density or spatial resolution. The LIFT technique offers high spatial resolution and high accuracy, and thus has high potential for the preparation of microarrays.

#### 5.1.1 Nucleic acid microarrays applications

Nucleic acid microarrays are a technique in which probes such as DNA, RNA and peptides are immobilised on the surface of a chip and hybridised to the labelled sample by the principle of base complementary pairing to achieve signal detection and analysis (Zhang et al., 2020). This technology has broad applications in drug screening, environmental monitoring, and disease diagnosis. Colina et al. successfully printed droplets containing cDNA onto substrates coated with L-polylysine using the LIFT technique, forming regular microarrays of deposited droplets with a diameter of 90 μm (the size could be smaller but was chosen for consistency with microneedle spotting) (Colina et al., 2005). By comparing the genetic signal of the DNA arrays with that of the microneedle-dotted samples, it was found that the genetic signal of the LIFT-printed DNA remained consistent with the microneedle-dotted samples, indicating that the laser pulses in LIFT caused little damage to the DNA. Other studies have also indicated that nucleic acid microarrays printed using LIFT technology yield comparable detection results to other conventional techniques and often offer higher printing resolution (Serra et al., 2004; Tsekenis et al., 2012; Tsekenis et al., 2015).

#### 5.1.2 Protein microarrays applications

**FIGURE 3:**
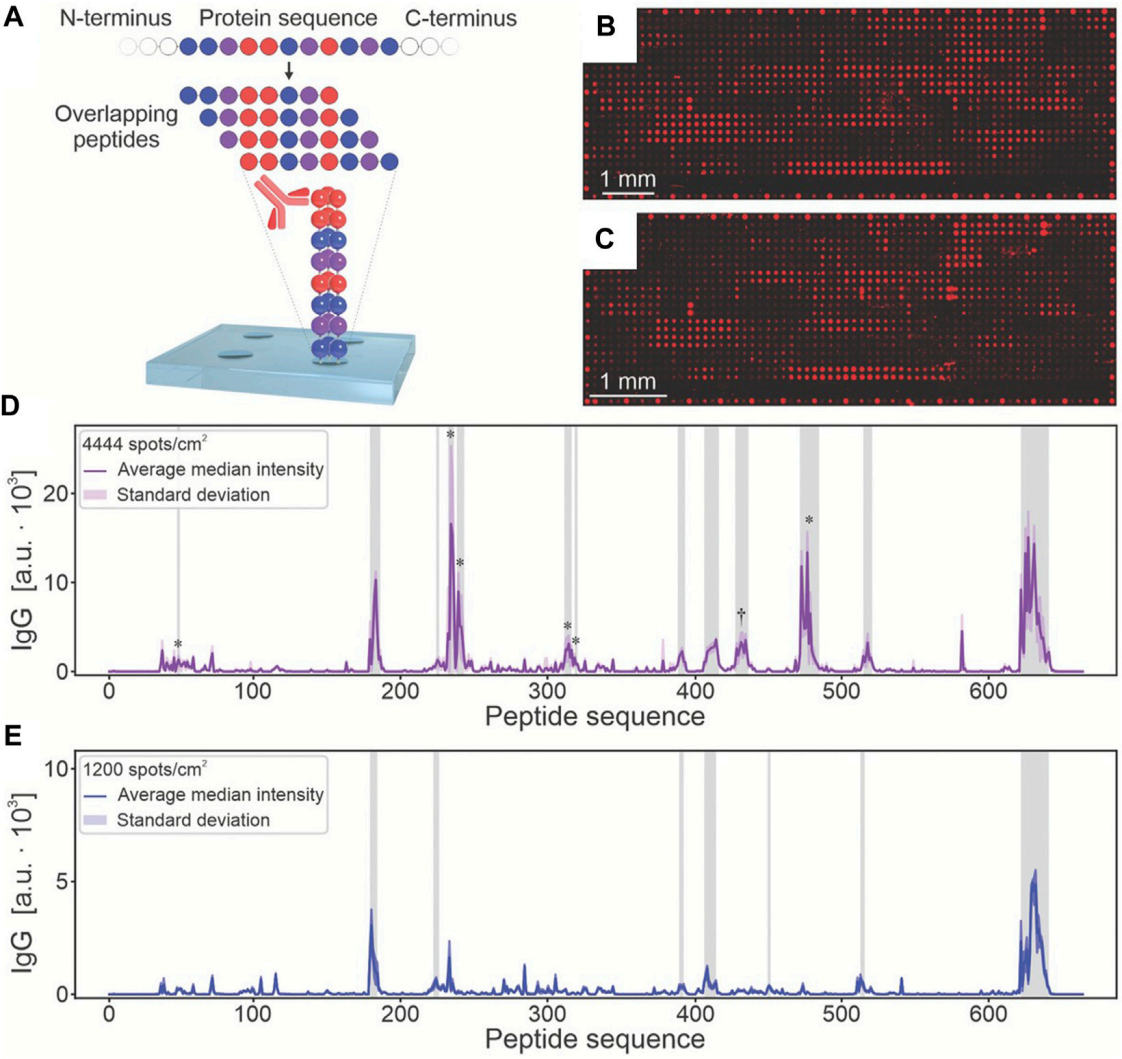
The microarray functionalized with Ebola virus surface glycoprotein peptide for screening IgG antibodies in disease survivors. **(A)**. The sequence of the Ebola virus surface glycoprotein was mapped into 662 overlapping 15-residue peptides, with a lateral shift of 1 amino acid per spot. After serum incubation, the binding of antibodies to arrays with **(B)** 4444 and **(C)** 10,000 spots/cm^2^ was analyzed by fluorescence imaging. The results signals of **(D)** 4444 spots/cm^2^ and **(E)** a commercial reference microarray were shown. (from ref. (Paris et al., 2022). licensed under Creative Commons Attribution 4.0 license).

Similar to nucleic acid microarrays, protein microarrays are a technology that involves immobilizing protein molecules (such as antibodies, antigens, enzymes, and receptors) as probes onto specially chemically treated substrates (Du et al., 2021; Wang et al., 2023). These probes’ specificity is utilized to detect target samples. Bovine serum albumin (BSA) was the first protein successfully printed using the LIFT technique from a biological solutions (Ringeisen et al., 2002). Subsequently, various peptides, proteins, antigens, and enzymes were also printed based on LIFT. Recently, Paris et al. (2022) proposed an automated synthesis instrument combining laser transmission and robotics for parallel synthesis in a microarray format with a spot density up to 10000 spots cm^−2^ which are much higher than commercial peptide arrays (Figure 3). The high-throughput microarray is used to analyze proteins from Ebola survivors. In comparison to the commercial Ebola virus surface glycoprotein microarray, this fabricated array not only displays existing epitopes but also detects additional epitopes known from the literature. These experiments demonstrate the ability of LIFT technology to print various proteins without disrupting their structure.

LIFT is also used for printing other biomaterials, such as lipid vesicles, which have been extensively studied as drug carriers and for applications in biosensing. However, lipid vesicles are molecular structures composed of lipid bilayers, and their diameters can reach several tens of micrometers, making them difficult to print using traditional direct printing techniques. Nevertheless, LIFT technology enables the printing of large-sized particles. Palla-Papavlu et al. (2011) successfully printed lipid vesicles using this technique, without causing damage to the vesicle membranes during printing.

### 5.2 LIFT printing of cells, tissues and organs

Human tissue is a complex three-dimensional structure composed of multiple types of cells and matrix components. The biomaterials surrounding cells in 3D cell printing can mimic the functionality of the extracellular matrix in the body, providing a suitable microenvironment for cell survival, proliferation, and differentiation (Kang et al., 2020; Mota et al., 2020; Wu et al., 2022). Most cell printing techniques have difficulty in meeting the high resolution (∼μm) required for the construction of microvasculature and nervous systems. LIFT technology allows for the accurate layer-by-layer deposition and stacking of active cells and biomaterials onto scaffolds in a defined spatial sequence, enabling the printing of various kinds of cells, tissues and organs.

#### 5.2.1 Cells applications

Cell printing serves as the foundation for tissue and organ printing, and many research groups have conducted studies on novel cell printing methods for tissue engineering. Bioinks containing cells, such as hydrogel solutions, typically have high viscosity, making them particularly suitable for printing using the LIFT technique. Moreover, LIFT is a non-contact and nozzle-free printing technology, which greatly reduces the issue of cross-contamination and to some extent eliminates clogging problems (Serra and Pique, 2019). The Chinese hamster ovaries eukaryotic cells were the first to be reported to be printed using LIFT technology, and no structural or genotype damage was observed in these cells (Wu et al., 2001). Ovsianikov et al. (2010) applied LIFT to introduce multiple cell types such as smooth muscle cells and ovine endothelial cells into the scaffold produced by two-photon polymerization technique, which may play an important role in 3D tissue printing with unprecedented flexibility and precision. Other types of cell such as human osteosarcoma cells (Barron et al., 2005), olfactory ensheathing cells (Othon et al., 2008) and neuronal cells (Patz et al., 2006) have also been reported to have been successfully printed using the LIFT technique. These printed cells typically maintain 100% viability and exhibit normal growth and proliferation without any functional or DNA damages. These findings demonstrate the feasibility and advantages of LIFT technology in cell printing, as it allows for the printing of high-viscosity bioinks and micrometer-scale cells while maintaining high printing resolution.

#### 5.2.2 Skin tissue applications

As the largest organ in the human body, the skin plays a crucial role in the body’s survival processes, providing functions such as barrier protection, immune response, prevention of water loss, and waste excretion. Severe extensive damage to the skin can result in life-threatening conditions for patients due to fluid loss, imbalanced osmotic pressure, and wound infections. Autologous skin grafts for patients with extensive skin damage often do not have sufficient skin donor sites, allogeneic and xenogeneic skin has immune rejection problems and skin wound excipients are not biologically active. 3D bioprinting technology offers the potential to custom manufacture functional skin substitutes, providing an effective solution to address the shortage of transplantable skin. Koch et al. (2012) conducted a 3D arrangement experiment using LIFT bioprinting to investigate the process of skin tissue formation. The study examined different parameters, including cell localization and proliferation, in a simplified model of double-layered skin tissue composed of fibroblasts and keratinocytes. The results demonstrated the potential and feasibility of bioprinting skin tissue. Michael et al. (2013) also conducted research on the development of skin substitutes using laser-assisted bioprinting technology. The skin construct consisted of 20 layers of fibroblasts and 20 layers of keratinocytes sequentially deposited onto Matriderm™ sheets. The effectiveness of the skin substitute was evaluated by transplanting it into skin wounds on the backs of mice. All mice in the experiment survived without showing any abnormal eating or sleeping patterns. Furthermore, the transplanted skin adhered to the wound edges after 11 days, and the presence of small blood vessels was observed. This study further confirms the potential of LIFT technology in skin bioprinting. Douillet et al. recently introduced the concept of “time” into 3D printing and proposed 4D bioprinting technology (Douillet et al., 2022). They utilized this technology to develop a new model for achieving dynamic remodeling of fibroblasts *in vitro*. This approach offers a versatile new tool for studying the characteristics of dermal tissue and its remodeling process during wound healing. LIFT bioprinting of skin currently enables the controlled printing of multi-layered skin substitutes. However, there are still significant challenges to be addressed before practical applications can be realized, such as the reconstruction of vascular networks, the construction of skin appendages, and the selection of suitable biomaterials.

#### 5.2.3 Blood vessels applications

**FIGURE 4:**
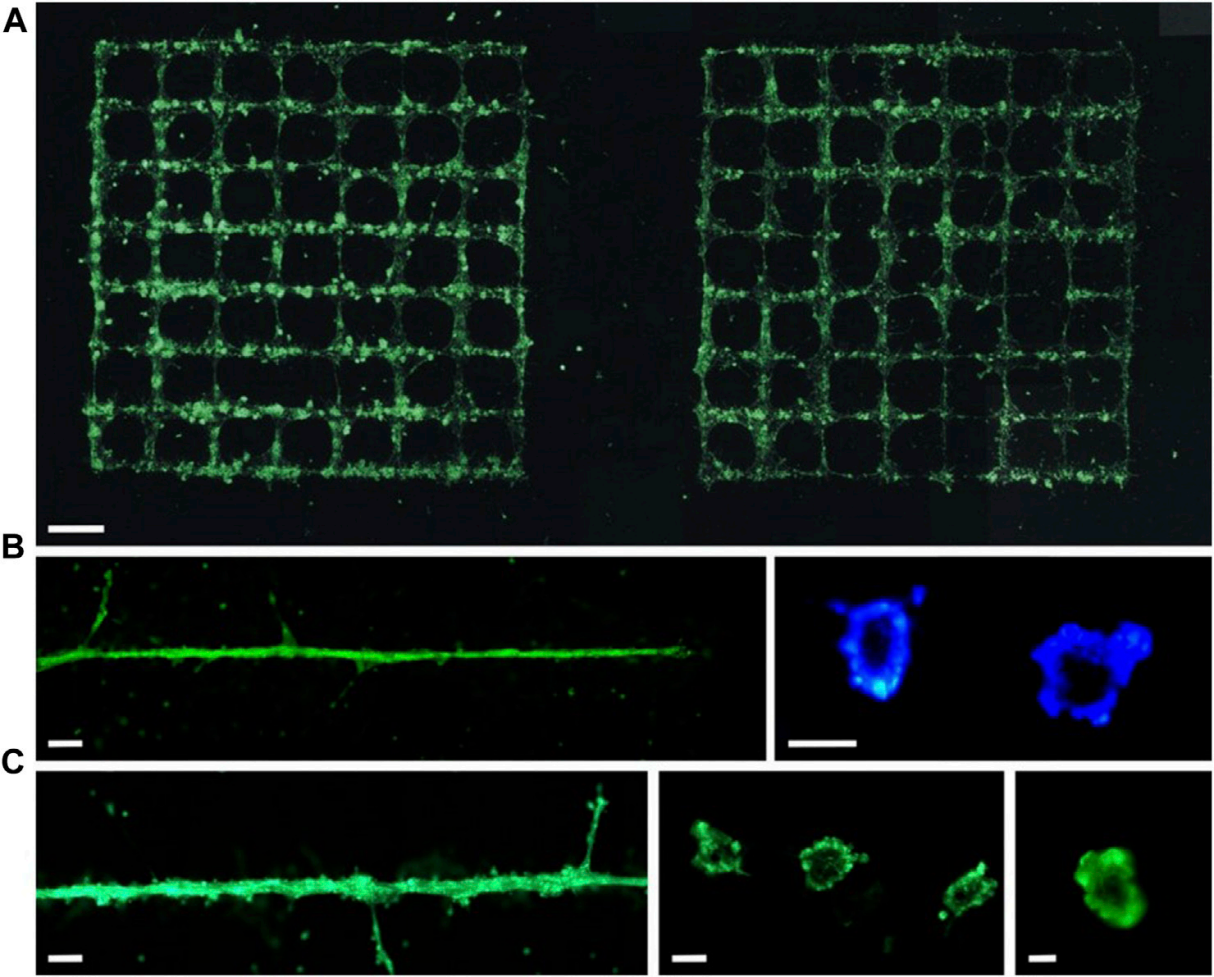
Vessels formed with labelled (green fluorescent) endothelial cells on Matrigel (within 24 h) are presented in **(A)** grids or **(B, C)** a line. Scale bars are 1 mm **(A)**, 200 µm [**(B)** left and **(C)** left], 50 µm [**(B)** right and **(C)** center], and 10 µm [**(C)** right]. (from ref. (Koch et al., 2021). licensed under Creative Commons Attribution 4.0 license).

Vascular diseases have always been one of the major medical challenges threatening human health. The current prevailing treatment method for such diseases is cardiovascular structure transplantation. However, the transplantation of biological grafts is associated with negative impacts including progressive occlusion, infection, limited regenerative potential, immune rejection reactions, and the risk of transmitting animal diseases. Moreover, the variations in anatomical structures among individuals result in diverse manifestations of cardiovascular defects, making the treatment of these diseases exceptionally challenging. In this regard, the emergence of 3D bioprinting technology enables the high-fidelity reconstruction of vascular structures and offers a relatively cost-effective solution. By precisely tailoring vascular substitutes based on individual patient characteristics, bioprinting minimizes transplantation risks and enhances regenerative potential. Xiong et al. (2015) achieved the printing of straight and Y-shaped tubes using two different bioinks (8% alginate solution and 2% alginate-based mouse fibroblast suspension) based on LIFT technology. After 24 h of incubation, the viability of the cells in the printed straight and Y-shaped tubes remained above 60%. The vascular network within tissues comprises blood vessels of varying diameters, with capillaries reaching sizes as small as 10 μm and there is a lack of reported studies on 3D bioprinting of capillaries. Koch et al. (2021) achieved the preparation of tubular structures of tens of micrometers in diameter using the LIFT 3D bioprinting technique (Figure 4). However, these printed capillaries lacked a natural structure and dissolved within 2 days after printing. Given the varying diameters of blood vessels within the human body, ranging from ∼10 μm to ∼1 mm, achieving proper integration between capillaries and larger blood vessels to ensure adequate blood perfusion poses a challenging task in tissue printing. This presents a formidable challenge for LIFT printing technology, which may necessitate the combination of other techniques to accomplish this objective.

#### 5.2.4 Other tissues or organs applications

**FIGURE 5:**
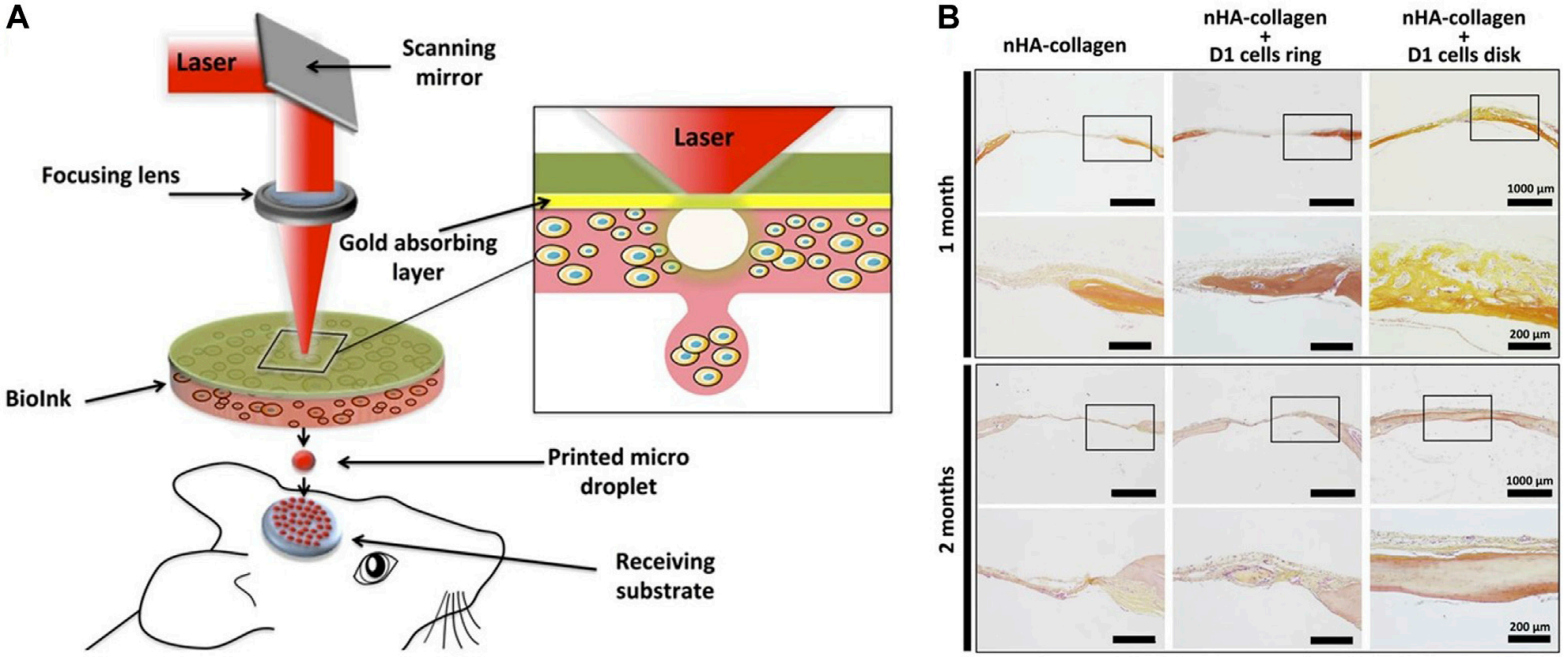
**(A)** Schematic of bone repair with the laser assisted bioprinting approach. **(B)** Histological evaluation of bone repair was performed through Hematoxylin/Eosin/Safran staining in mouse tibial defects at 1 and 2 months post bioprinting. [from ref. (Keriquel et al., 2017). licensed under Creative Commons Attribution 4.0 license].

LIFT-based 3D bioprinting technology has also been utilized for the printing of other tissue and organ structures, including the bone, neural structures and the heart (Gaebel et al., 2011; Curley et al., 2016). These applications highlight the immense potential of this technology in the field of tissue engineering. Catros et al. utilized laser printing to create 2D and 3D composite structures by printing human osteoprogenitors derived from human bone marrow stromal cells (Catros et al., 2011a). The resulting structures demonstrated favorable functionality and maintained the properties of cell proliferation and differentiation after the bioprinting process. Keriquel et al. (2017) utilized LIFT technology for *in situ* printing of mesenchymal stromal cells, associated with collagen and nano-hydroxyapatite, to investigate bone regeneration in a mouse calvaria defect model (Figure 5). By testing different cell printing geometries, the results demonstrated the impact of varying cellular arrangements on bone tissue regeneration. Curley et al. (2016) utilized laser printing technology to print dorsal root ganglion neurons, and the printed cells maintained a high level of vitality, exhibiting neuronal processes capable of nerve growth and network formation. Moreover, they demonstrated the expected migration and proliferation of the cells. Due to the ability to self-renew, similar to embryonic stem cells, human induced pluripotent stem cells (iPSCs) can undergo extensive expansion and maintain the potential to differentiate into all cell types of the human body (Takahashi et al., 2007; Yu et al., 2007). Therefore, these cells have been widely applied in 3D bioprinting. Koch et al. (2018) successfully demonstrated the laser printing of hiPSCs by selecting appropriate bioink materials. They found that iPSCs were more sensitive to the bioink materials, and the key to successful iPSC printing lied in the selection of suitable biomaterials such as hydrogels and sols, which enabled the differentiation towards cardiomyocytes. Gaebel et al. (2011) applied the LIFT technology to print human umbilical vein endothelial cells and human MSC on a Polyester urethane urea cardiac patch for cardiac regeneration. The results demonstrated that the LIFT-printed patches facilitated the formation of blood vessels when transplanted into RNU rats and could significantly improve the function of their infarcted hearts.

## 6 Future perspective and conclusion

With its advantages of high precision, accuracy, and cell viability, LIFT technology has found extensive application in various biomedical fields, including drug delivery and testing, nucleic acid microarray printing and tissue bioprinting. However, several technical challenges need to be addressed before LIFT-based bioprinting products become commonplace. These challenges are discussed from the following perspectives:

1. The quality of laser printing is directly determined by the dynamics of jet formation. Therefore, it is crucial to clarify the physics processes underlying bubble and jet formation. In order to accurately predict the initial bubble formation and the jetting behavior under various printing conditions, it is necessary to continuously verify and optimize different theories, thereby establishing an accurate physical model. The formation of bubbles and jets involves high pressure, high acceleration, and rapid temperature changes. In-depth research on this topic can further optimize the process of bioprinting.
2. Creating realistic tissue organs involves different types of cells with varying shapes, making it a challenge to select suitable printing parameters. LIFT printing is characterized by slow printing speed, and when printing under ambient conditions, the properties of the bio-ink, such as viscosity and cell density, tend to change over time. This can significantly impact the printing quality of tissue organs. Therefore, real-time monitoring of the ink’s condition to select appropriate printing parameters is necessary, or appropriate measures should be taken to prevent ink from drying during the printing process.
3. The obstacles between laboratory research findings and commercial production are significant. The significant differences between laboratory research and commercial production include scale, technological requirements, standardization, validation, and financial aspects. Currently, tissue printing in laboratory research is still immature. Thus it is necessary to further enhance the stability, success rate, and repeatability of printing to improve feasibility. Meanwhile, limited by the current speed of LIFT printing, it is not feasible to manufacture bio-products on a large scale with high efficiency. Therefore, optimization of experimental equipment and processes is required to achieve high-speed, high-efficiency, and cost-effective large-scale biomanufacturing.

In summary, LIFT technology has garnered widespread attention since its application in the biomedical field. The feasibility of printing biomolecules and cells using LIFT has been demonstrated in numerous studies. This review provides an overview of the concept, jetting mechanism, and relevant printing parameters of LIFT. It also discusses the biomedical applications of LIFT in printing cells (osteosarcoma, endothelial and mesenchymal stem cells) or tissues (skin, blood vessels, bone and neural tissues). Research focusing on LIFT bioprinting is still limited due to the complexity of implementing LIFT technology and the higher cost of equipment. However, with advancements in related technologies such as biology, materials, and automation, it is anticipated that more researchers will be attracted to engage in LIFT bioprinting.
